# Maternal anxiety and depression and their associations with mother–child pretend play: a longitudinal observational study

**DOI:** 10.1186/s40359-021-00568-9

**Published:** 2021-05-07

**Authors:** Zhen Rao, Beth Barker, Christine O’Farrelly, Paul Ramchandani

**Affiliations:** 1 Centre for Research On Play in Education, Development and Learning, Faculty of Education, University of Cambridge, 184 Hills Road, Cambridge, CB2 8PQ UK; 2 Division of Psychiatry, Imperial College London, Du Cane Road, London, W12 0NN UK

**Keywords:** Anxiety, Depression, Mother–child interaction, Pretend play, Behavioural problem

## Abstract

**Background:**

Parental anxiety and depression have been associated with changes to parent–child interactions. Although play constitutes an important part of parent–child interactions and affords critical developmental opportunities, little is known regarding how parental anxiety and depression are related to parent–child play. This is an important knowledge gap because parents play a crucial role in children’s early play experience. The purpose of the current study was to examine whether levels of maternal anxiety and depression respectively predicted frequencies of pretend play in both mothers and their children, and whether mothers’ engagement in pretend play predicted child behaviour problems two years later.

**Methods:**

Pretend play in 60 mother-toddler dyads (*M*_age of child_ = 29.67 months*, SD* = 3.25*,* 41.7% girls) was assessed during home visits. Maternal anxiety and depression were assessed using self-report questionnaires. Children’s behaviour problems were rated by mothers at baseline and two years later. Hierarchical regression analyses examined concurrent associations between mother–child pretend play and maternal anxiety and depression at baseline, and longitudinal associations between baseline mother pretend play and child behavioural problems two years later.

**Results:**

Higher maternal anxiety predicted less pretend play in mothers and children (β =  − .23, BCa 95% CI: [− .018, − .001]) and β =  − .22, BCa 95% CI [− .014, − .001]). Higher maternal depression predicted less child pretend play (β =  − .20, BCa 95% CI [− .012, − .001]). There was evidence (albeit weak) that more mother pretend play at baseline predicted fewer child behaviour problems two years later (β =  − .18, BCa 95% CI [− 62.38, 11.69]), when baseline child behaviour problems and maternal anxiety were controlled for.

**Conclusions:**

Maternal anxiety and depression are associated with less pretend play during mother–child interaction. Mother’s pretend play might help reduce child behavioural problems risks, suggesting that play might be one mechanism by which maternal mental health influences children’s development.

## Background

Anxiety and depression are common mental health problems faced by parents [6, 23]. In England, for example, around one in six adults aged 16 to 64 years have a common mental health disorder including anxiety or depression, and around one in three children live with at least one parent reporting symptoms of emotional distress [25]. Research has suggested that parental anxiety and depression are associated with changes to parent–child interactions and adverse child outcomes [12, 14, 19]. To understand the mechanisms behind this, previous research has examined various aspects of positive (e.g., autonomy granting, warmth, sensitivity, responsiveness, acceptance, and encouragement) and negative (e.g., control, overprotection, intrusiveness, withdrawal, criticism, and harshness) parenting behaviours [10, 19] in relation to parental anxiety and depression. Despite being an essential part of parent–child interaction, parent–child play has mostly been used as a context to examine the above aspects of parenting behaviours (e.g., whether parents are sensitive or intrusive during play interaction with their children), rather than being investigated in its own right (e.g., whether parents engage in specific types of play such as pretend play or physical play) in relation to parental anxiety and depression. This is an important gap because play has a critical role in promoting children’s cognitive, physical, social, and emotional wellbeing and building positive parent–child relationships [11]. Additionally, parental play behaviour has features (e.g., non-literalness, novelty, flexibility, ambiguity, uncertainty, and humour) that distinguish it from other aspects of parenting behaviours (e.g., feeding, soothing, physical caretaking, and protection) [30, 33]. In this way parental play may provide important developmental opportunities (e.g., allowing children to try out different emotional regulation strategies without serious consequences) that non-play parenting behaviours (e.g., caregiving and discipline) might not afford, in addition to being a fun and engaging context for parent–child interaction. Examining play as a dimension of parenting behaviour, therefore, has unique value in furthering our understanding of the pathways through which parental anxiety and depression affect parenting behaviours and child development. 

As an important form of parent–child play, pretend play has been defined as playful activities that include at least one of the three fundamental forms of pretence (i.e., object substitution, pretend attribution of properties, and imaginary objects [16]). Being performed in a non-literal way, pretend play has been conceptualised as providing a relatively safe context for individuals to explore experiences and emotions, because the risks of such exploration are potentially reduced in the pretend context compared to real situations [4, 8]. Such a safe context is especially important for exploring negative emotions (e.g., fear and worries) and challenging experiences (e.g., risks and conflicts) with social partners, which could provide children with opportunities to develop emotional regulation and social skills [3, 35]. The deliberate modification of behaviour during pretend play (e.g., drinking from an empty cup) and the associated incongruity (e.g., the mismatch between the drinking action and the fact that there is nothing real to drink) could also serve as positive social signals to play partners around how to interpret the interaction (e.g., “Let’s not take things seriously”), as well as providing a context for experiencing humour and positive emotions [2]. When engaging in pretend play with their children, parents thus provide their children with the opportunities to experience positive emotions, to regulate negative emotions in a safer context, and to develop social and communication skills [34]. An increasing body of empirical evidence has also suggested that engagement in pretend play may be closely related to children’s emotional experience, regulatory skills and behavioural problems [28].

Although pretend play may provide important opportunities for children’s social and emotional development, research has also suggested that emotional difficulties may hinder children’s engagement in pretend play. For example, preschool girls with lower emotional regulation ability were found to pretend less frequently than those with higher emotion regulation ability [18]. Children with depression aged 3 to 6 years were found to show significantly less pretend play than non-depressed children [22]. As pretend play can evoke emotions of varying intensity, the players are required to keep these emotional arousals at appropriate levels in order for the play to continue [9]. An inability to regulate these emotional arousals, therefore, may hinder one’s engagement in pretend play, which could have potentially benefited their emotional wellbeing.

Closely related to children’s (especially young children’s) engagement in pretend play is the important role that parents have in facilitating this form of play. Because their language, cognitive, social, and emotional abilities are under development, young children’s engagement in pretend play is commonly facilitated by their parents. Research has shown that mothers may initiate and model pretend play, suggest pretend ideas, and extend young children’s pretend scenarios by providing cues (e.g., exaggerated behaviour, varied pitch, extra sound effects, longer looking at the child, more frequent smiles and laughter) to help young children to understand pretend play [17].

This important role that mothers play in facilitating their young children’s pretend play, however, may be negatively impacted by maternal anxiety and depression. Evidence has shown that maternal mood can affect mother–child interaction in a variety of ways (e.g., [14, 23]. Being caught up in negative thoughts and/or experiencing interpretation biases, anxious or depressed mothers may have difficulty noticing and correctly interpreting their child’s signals, making them less likely to respond appropriately to their child and provide support [23]. Additionally, they may be withdrawn or intrusive, show less affection, and/or less vocalisation when interacting with their child [14, 32]. These potential impacts on mother–child interaction are likely to have implications on how mothers engage in pretend play with their child. For instance, mothers may be less likely to notice their child’s pretend signals, and more likely to interpret the ambiguity, novelty, and uncertainty embedded in pretend play in negative ways [20]. They may find it difficult to use exaggerative tones or behaviour to initiate and maintain pretend play, or to use smiles and laughter to signal play. Their emotional vulnerabilities could also make it difficult for these mothers to cope with the emotional arousal evoked by pretend scenarios. In addition to directly restricting their own and their children’s engagement in play, anxious or depressed mothers may indirectly influence their children’s play through the mother–child relationship. Evidence has suggested that mothers of securely attached children were more involved in their children’s play [29], and securely attached children were better able to incorporate an experimenter’s suggestions into their pretend play [21].

Despite the evidence that maternal anxiety and depression may lead to diminished quality of mother–child interaction, little is known about the relationship between maternal mental health and mother–child pretend play. This is an important knowledge gap that needs to be addressed. As discussed above, pretend play provides significant opportunities for children to develop their emotional regulation and social skills. Understanding how mother–child pretend play might be affected by maternal anxiety and depression would help to identify factors that may facilitate or hinder children in their social and emotional development through pretend play. This could have important implications for future interventions aiming to promote positive mother–child interactions.

Additionally, it is important to investigate the potential impacts of anxious and depressed mothers’ engagement in pretend play on their child’s behavioural outcomes. Despite accumulating evidence that maternal anxiety and depression could increase the risks of child behaviour problems [10, 12], no previous research has examined how pretend play might be implicated in this association. Given the role of pretend play in facilitating the development of emotional regulation and social skills, it is possible that mother–child pretend play serves as a factor to reduce child behavioural problem risks. Understanding this could have implications for future interventions aiming to prevent the development of behaviour problems in children of anxious or depressed parents.

### The current study

As an attempt to address the knowledge gaps discussed above, the current study examined whether mother–child pretend play was associated with maternal anxiety and depression concurrently, and associated with child behaviour problems longitudinally. Pretend play was examined during a five-minute interaction between 60 mothers and their 2-year-old children recorded at home. Maternal anxiety and depression were assessed by self-report questionnaire. Children’s behaviour problems were rated by mothers at baseline and two years later. The current study aimed to test three hypotheses: (1) Higher maternal anxiety will predict less pretend play in both mothers and their children. (2) Higher maternal depression will predict less pretend play in both mothers and their children. (3) More mother pretend play will predict less child behaviour problem two years later over and above baseline child behaviour problems and maternal anxiety and depression. The first two hypotheses aimed to address the knowledge gap on how maternal anxiety and depression may negatively affect mother and child pretend play. The third hypothesis aimed to address the knowledge gap on whether mother’s engagement in pretend play may reduce the risks of child behaviour problem associated with maternal anxiety and depression.

## Methods

### Participants

The participants comprised of 60 mother–child dyads (*M*_age of child_ = *29.67 months, SD* = *3.25, 41.7%* girls) who took part in a large psychological intervention trial [24, 26]. The trial involved 300 children aged 12–36 months who were at risk of behavioural difficulties (i.e., scored in the top 20% for behavioural problems on the Strengths and Difficulties Questionnaire; [13] and their parents. Parents were recruited predominantly through UK National Health Service (NHS) health visiting services, either face-to-face during routine child health reviews or through targeted mailshots. Families were also recruited through wider clinical and community services including children’s centres, child and adolescent mental health services, GP services, and advertisements in community settings and online groups. Written informed consent was obtained from the parents at each phase of the study. The trial was reviewed by the National Research Ethics Service (NRES) Committee London – Riverside Research Ethics Committee (REC reference: 14/LO/2071). All methods were performed in accordance with the relevant guidelines and regulations.

The 300 families were randomly allocated into an intervention group or a control group following a baseline assessment. The current study involved all children from the control group aged between 24 and 36 months at the baseline home visit, where the mother–child dyads (*N* = 60 dyads) were provided with the same toy set. Mother’s ethnicities were as follows: 68.3% White, 13.3% Mixed, 11.7% Black, 3.3% Asian and 3.3% Other. The sample was relatively highly educated: 65% of mothers held a graduate level qualification, 23.3% held a post-secondary qualification, and 11.7% held qualifications at GCSE level or lower. The age range of the children was selected because pretend play typically emerges around 18 months and is generally present by age 2 [17].

### Procedures

Data used for the current study were collected by trained researchers during home-based assessments at two time points: one visit at baseline and one visit at two-year follow-up (24 months after enrolment into the study). During the baseline visit, a 5-minute mother–child free play interaction was videotaped for each family. During the play interaction parents were presented with a bag of toys and asked to play as they normally would. Parents were asked to only play with the provided toys (rather than the child’s own toys). At both the baseline and the two-year follow-up visits, parents were also asked to complete a series of questionnaires including measures of child behaviour and their own anxiety and depression.

### Measures

#### Mother–child pretend play

Mother–child pretend play was examined by coding a video-recorded play interaction between the mother–child dyad during the baseline home visit. A bag containing a toy picnic set, two hand puppets and a car was given to the dyad and parents were encouraged to play with their child as they ‘normally would’ using the toys provided. The mother–child interaction was video-recorded for five minutes by the researcher using a hand-held camera.

Videos of mother–child play interaction were later analysed at five-second segments using the software ELAN (2017). Researchers who coded the videos were not involved in data collection and were blind to family characteristics when conducting the coding. For each five-second segment of the videos, the code “mother pretend” was assigned if the mother exhibited any pretend play behaviour during the segment. The code “mother non-pretend” was assigned if the mother did not exhibit any pretend play during the segment. Likewise, the code “child pretend” or “child non-pretend” was assigned to each segment depending on whether the child exhibited any pretend behaviour (e.g., pouring the toy teapot, drinking from an empty cup. See Appendix for the detailed coding scheme). Reliability coding was conducted with an independent coder based on time-aligned segments from 19 randomly selected videos (32% of 60 videos). Cohen’s Kappa for parent pretend and child pretend were 0.8 and 0.825, respectively. A child (or mother) pretend play score was obtained by dividing the total number of segments coded as “child pretend” (or “mother pretend”) for each child (or mother) by the total number of segments coded for the child (or mother).

#### Maternal anxiety

Maternal anxiety was measured using the Generalised Anxiety Disorder 7 (GAD-7) [31], a seven-item questionnaire that has been widely used in research as a general measure of anxiety in adults. Mothers were asked how often they were bothered by each problem (“not at all” = 0, “several days” = 1, “more than half the days” = 2, or “nearly every day” = 3) during the last two weeks. A total score was obtained by summing all items, with severity ranging from minimal (i.e., 0–4), to mild (i.e., 5–9), moderate (i.e., 10–14), and severe (i.e., 15–21).

#### Maternal depression

Maternal depression was measured using the Patient Health Questionnaire 9 (PHQ-9), a nine-item questionnaire that has been widely used to index depression severity [15]. Each item was scored on the frequency of a problem experienced by the mothers over the past two weeks (“not at all” = 0, “several days” = 1, “more than half the days” = 2, or “nearly every day” = 3). A total score was obtained by summing all items of the questionnaire and ranged from 0 to 27, with 5, 10, 15, and 20 representing the lower limits of mild, moderate, moderately severe, and severe depression.

#### Child behaviour problems at baseline and 2-year follow-up

Child behaviour at baseline and at 2-year follow-up were measured by the Child Behaviour Checklist/1½—5 (CBCL/1½-5; [1]), a widely used questionnaire that has been standardised with children aged 18 months to 5 years old. The questionnaire included 100 items measuring a broad range of behavioural problems young children may exhibit. Mothers were asked to rate the degree to which they believe it was true for the past two months, with each item scored 0 (not true), 1 (somewhat/sometimes true), or 2 (very/often true). The total CBCL score was used for the current study.

### Planned analysis

There were three hypotheses for the current study. The first hypothesis was that higher maternal anxiety level would predict lower frequency of both mother and child pretend play. The second hypothesis was that higher maternal depression level would predict lower frequency of both mother and child pretend play. A linear regression model using maternal anxiety score (for the first hypothesis), or maternal depression score (for the second hypothesis) to predict mother pretend play frequency would be first tested. Hierarchical linear regression would then be tested using maternal anxiety score (for the first hypothesis), or maternal depression score (for the second hypothesis) to predict child pretend play frequency, controlling for mother pretend play frequency.

The third hypothesis was that higher frequency of mother pretend play at baseline would predict fewer child behavioural problems at 2-year follow-up, controlling for baseline child behavioural problems, baseline maternal anxiety and depression scores. Hierarchical linear regression would be used for testing this hypothesis. If mother pretend play significantly predicted child behavioural problems, the interactions between mother pretend play and baseline child behavioural problems, maternal anxiety and/or depression would be tested.

## Results

### Descriptive statistics

Statistical analyses were conducted using SPSS. As can be seen from Table 1, mothers on average engaged in pretend play more frequently (28% compared to 18% of the 60 five-second segments) than their children during the five-minute interaction. A strong correlation was observed between the frequencies of mother pretend play and child pretend play (*r* (58) = 0.71, *p* < 0.001). Neither mother pretend play nor child pretend play frequency was significantly correlated with child age. No significant difference was observed in child or mother pretend play frequency in terms of child gender (*t* (58) =  − 0.40, *p* = . 688 and *t* (58) =  − 0.44, *p* = 0.665 respectively). No gender difference was observed in behavioural problems at 2-year follow-up (*t* (54.61) =  − 0.70, *p* = 0.487).

**Table 1:** Means, standard deviations, and correlations

Variables	*n*	*M*	*SD*	1	2	3	4	5	6	7
1. Mother pretend^ab^	60	0.28	0.16							
2. Child pretend^a^	60	0.18	0.13	.71^**^						
3. Maternal anxiety	60	5.06	3.87	− .23^*c^	− .37^** c^					
4. Maternal depression^b^	60	4.88	4.75	− .15^c^	− .30^*c^	.55^**c^				
5. Child baseline age in month	60	29.67	3.25	.14	.11	.09	.05			
6. Child baseline CBCL	60	46.06	23.82	− .07	− .20	.46^**^	.52^**^	.27^*^		
7. Child CBCL 2 years later^b^	57	34.87	23.26	− .32^* d^	− .38^** d^	.54^** d^	.49^** d^	.04	.65^**^	

*Note. CBCL*, Child Behaviour Checklist

^a^Score represents the percentage of five-second segments an individual was observed engaging in pretend play out of 60 segments (5 min)

^b^Score of one univariate outlier was replaced by the next highest score plus one unit

^c^Correlations were calculated on 59 mothers with one binary outlier removed

^d^Correlations were calculated on 56 children with one binary outlier removed

^*^ *p* < .05. ** *p* < .01

A small negative correlation was observed between mother pretend play and maternal anxiety score (*r* (57) =  − 0.23, *p* = 0.041, one-tailed), whereas no evidence was seen for an association between mother pretend play and maternal depression scores (*r* (57) =  − 0.15, *p* = 0.253). Child pretend play was negatively correlated with maternal anxiety and depression, with similar moderate effect sizes (*r* (57) =  − 0.37, *p* = 0.004 and *r* (57) =  − 0.3, *p* = 0.023, respectively). Child CBCL score at 2-year follow-up^1^ was positively correlated with maternal anxiety and depression with moderate to large effect sizes (*r* (54) = 0.54 and 0.49 respectively, *p*s < 0.001), and negatively correlated with the frequencies of mother pretend play and child pretend play with moderate effect sizes (*r* (54) =  − 0.32, *p* = 0.017 and *r* (54) =  − 0.38, *p* = 0.004, respectively).

### Hypothesis 1: maternal anxiety predicting mother and child pretend play

A linear regression was first conducted to test the hypothesis that higher maternal anxiety level predicted lower frequency of mother pretend play. Bootstrap results showed that maternal anxiety score negatively predicted mother pretend play score (*R*^2^ = 0.05, *F* (1, 57) = 3.23, Β =  − 0.01, SE = 0.004, β =  − 0.23, *p* = 0.02, BCa 95% CI: [− 0.018, − 0.001]). This indicated that mother pretend play decreased 1% for every unit of increase in maternal anxiety score.

Next, a hierarchical linear regression was conducted to test the hypothesis that higher maternal anxiety level predicted lower frequency of child pretend play, controlling for the frequency of mother pretend play (Table 2). The first model using mother pretend play to predict child pretend play was significant (*R*^2^ = 0.51, *F* (1, 57) = 58.52, *p* < 0.001). The second model adding maternal anxiety as a predictor was significant (*R*^2^ = 0.55, *F* (1, 56) = 5.51, *p* = 0.022). This model indicated that child pretend play was positively predicted by mother pretend play (Β = 0.54, *SE* = 0.08, β = 0.66, *p* < 0.001, 95% CI [0.39, 0.69], BCa 95% CI [0.37, 0.69]) and negatively predicted by maternal anxiety (Β =  − 0.01, *SE* = 0.003, β =  − 0.22, *p* = 0.022, 95% CI [− 0.01, − 0.001], BCa 95% CI [− 0.01, − 0.001]). Child pretend play decreased 1% for every unit of increase in maternal anxiety.

**Table 2:** Regression results for predicting child pretend play using mother pretend play and maternal anxiety

Variables	B	95% CI		SE	*p*	β	R^2^	∆R^2^
		LL	UL					
*Model 1*							.51	.51
Constant	.02	− .03	.07	.02	.429			
Mother pretend play	.58	.43	.73	.08	< .001	.71		
*Model 2*							.55	.04
Constant	.07	.01	.13	.03	.034			
Mother pretend play	.54	.39	.69	.08	< .001	.66		
Maternal anxiety	− .01	.003	− .01	− .001	.022	− .22		

*N* = 59. One binary outlier was excluded from the regression. *CI*, confidence interval; *LL*, lower limit; *UL*, upper limit

### Hypothesis 2: maternal depression predicting mother and child pretend play

A linear regression was first conducted to test the hypothesis that higher maternal depression level predicted lower frequency of mother pretend play. Bootstrap results showed that maternal depression score did not significantly predict mother pretend play score (*R*^2^ = 0.02, *F* (1, 57) = 1.19, Β =  − 0.01, SE = 0.005, β =  − 0.14, *p* = 0.231, BCa 95% CI: [− 0.014, 0.007]).

Next, a hierarchical linear regression was conducted to test the hypothesis that maternal depression negatively predicted the frequency of child pretend play, controlling for the frequency of mother pretend play (Table 3). The first model using mother pretend play to predict child pretend play was significant (*R*^2^ = 0.47, *F* (1, 57) = 49.67, *p* < 0.001). The second model adding maternal depression as a predictor was significant (*R*^2^ = 0.51, *F* (1, 56) = 4.51, *p* = 0.038). This model indicated that child pretend play was positively predicted by mother pretend play (Β = 0.52, *SE* = 0.08, β = 0.65, *p* < 0.001, 95% CI [0.37, 0.68], BCa 95% CI [0.40, 0.66]) and negatively predicted by maternal depression (Β =  − 0.01, *SE* = 0.003, β =  − 0.20, *p* = 0.038, 95% CI [− 0.012, − 0.0004], BCa 95% CI [− 0.012, − 0.001]). Child pretend play decreased 1% for every unit of increase in maternal depression.

**Table 3:** Regression results for predicting child pretend play using mother pretend play and maternal depression

Variables	B	95% CI		SE	*p*	β	R^2^	∆R^2^
		LL	UL					
*Model 1*							.47	.47
Constant	.03	− .02	.08	.02	.283			
Mother pretend play	.55	.39	.70	.08	< .001	.68		
*Model 2*							.51	.04
Constant	.06			.03	.038			
Mother pretend play	.52	.37	.68	.07	< .001	.65		
Maternal depression	− .01	− .01	− .0004	.003	.038	− .20		

*N* = 59. One binary outlier was excluded from the regression. *CI*, confidence interval; *LL*, lower limit; *UL*, upper limit

### Hypothesis 3: Mother pretend play predicting child behaviour at 2-year follow-up

A hierarchical linear regression was performed to test the third hypothesis that more mother pretend play at baseline predicted lower CBCL scores at 2-year follow-up, controlling for baseline CBCL, baseline maternal anxiety, and baseline maternal depression (Table 4).

**Table 4:** Regression results for predicting chid behavioural problem at 2-year follow-up

Variables	B	95% CI		SE	*p*	β	R^2^	∆R^2^
		LL	UL					
*Model 1*							.46	.46
Constant	5.30	− 4.53	15.12	4.89	.284			
Baseline CBCL	.43	.20	.66	.12	< .001	.46		
Baseline maternal anxiety	1.38	− .20	2.95	.79	.085	.25		
Baseline maternal depression	.39	− 1.17	1.95	.78	.617	.07		
*Model 2*							.46	.46
Constant	5.19	− 4.55	14.92	4.86	.29			
Baseline CBCL	.45	.23	.67	.11	< .001	.48		
Baseline maternal anxiety	1.60	.31	2.90	.65	.016	.29		
*Model 3*							.49	.03
Constant	14.19	.37	28.00	6.89	.044			
Baseline CBCL	.41	.20	.63	.11	< .001	.45		
Baseline maternal anxiety	1.56	.29	2.82	.63	.017	.28		
Baseline mother pretend play	− 26.08	− 55.05	2.88	14.43	.077	− .18		

*N* = 56. Three children from the baseline sample who did not complete the 2-year follow up and one multivariate outlier were excluded for the testing of this hypothesis. *CBCL*, Child Behaviour Checklist; *CI*, confidence interval; *LL*, lower limit; *UL*, upper limit

The first model using the three control variables to predict child CBCL at 2-year follow-up was significant (*R*^2^ = 0.46, *F* (3, 52) = 14.78, *p* < 0.001). This model indicated that baseline CBCL significantly predicted CBCL at 2-year follow-up (Β = 0.43, *SE* = 0.12, β = 0.46, *p* < 0.001, 95% CI [0.20, 0.66], BCa 95% CI [0.14, 0.69]). There was evidence (albeit weak) that higher baseline maternal anxiety predicted higher CBCL score at 2-year follow-up (Β = 1.38, *SE* = 0.79, β = 0.25, *p* = 0.085, 95% CI [− 0.20, 2.95], BCa 95% CI [− 0.72, 3.01]). Baseline maternal depression did not significantly predict CBCL at 2-year follow-up (Β = 0.39, *SE* = 0.78, β = 0.07, *p* = 0.617, 95% CI [− 1.17, 1.95], BCa 95% CI [− 1.86, 2.34]). Although baseline CBCL, maternal anxiety and depression were correlated, no multicollinearity was observed.

The second model dropped baseline maternal depression and used baseline CBCL and baseline maternal anxiety to predict CBCL at 2-year follow-up. This model was significant (*R*^2^ = 0.46, *F* (2, 53) = 22.36, *p* < 0.001) and indicated that CBCL at 2-year follow-up was significantly predicted by baseline CBCL (Β = 0.45, *SE* = 0.11, β = 0.48, *p* < 0.001, 95% CI [0.23, 0.67], BCa 95% CI [0.22, 0.65]) and baseline maternal anxiety (Β = 1.60, *SE* = 0.65, β = 0.29, *p* = 0.016, 95% CI [0.31, 2.90], BCa 95% CI [0.34, 3.14]).

The third model added baseline mother pretend play in addition to baseline CBCL and maternal anxiety in the second model as predictors. The third model indicated that CBCL at 2-year follow-up was significantly predicted by baseline CBCL (Β = 0.41, *SE* = 0.11, β = 0.45, *p* < 0.001, 95% CI [0.20, 0.63], BCa 95% CI [0.15, 0.64]) and maternal anxiety (Β = 1.56, *SE* = 0.63, β = 0.28, *p* = 0.017, 95% CI [0.29, 2.82], BCa 95% CI [0.37, 3.08]). There was evidence (albeit weak) that more baseline mother pretend play predicted lower CBCL score at 2-year follow-up (Β =  − 26.08, *SE* = 14.43, β =  − 0.18, *p* = 0.077, 95% CI [− 55.05, 2.88], BCa 95% CI [− 62.38, 11.69]). This model indicated that child CBCL score at 2-year follow-up decreased 0.26 for each 1% of increase in baseline mother pretend play, when baseline CBCL and baseline maternal anxiety were controlled for. Testing for the interactions between baseline mother pretend play, baseline CBCL, and baseline maternal anxiety indicated no significant two-way (*p* = 0.453, 0.299 and 0.681 respectively) or three-way (*p* = 0.404) interactions. This suggested that the relationship between baseline mother pretend play and child behavioural problems at 2-year follow-up did not differ according to baseline CBCL and baseline maternal anxiety.

## Discussion

The purpose of the current study was to examine whether higher levels of maternal anxiety and depression respectively predicted less pretend play in both mothers and their children, and whether mothers’ higher engagement in pretend play predicted fewer child behaviour problems two years later. It is important to investigate these questions, because mothers play a crucial role in their child’s early pretend play experience, which affords critical opportunities for children’s cognitive, social, and emotional development. The current study found that compared with mothers with lower anxiety levels, those with higher anxiety levels were less likely to engage in pretend play when interacting with their children. This suggests that anxious mothers may have difficulty in engaging in pretend play when interacting with their children. For example, they may have difficulty noticing or correctly interpreting children’s pretend signals, or generating pretend ideas [20]. When mothers’ engagement in pretend play was controlled for, the current study found that children of mothers with higher anxiety or depression levels were less likely to engage in pretend play, with a small effect size. This indicates that the negative impact of maternal anxiety and depression on child pretend play behaviour may exist beyond reducing mother pretend play behaviour. For example, anxious or depressed mothers may discourage their children from pretending via modelling (e.g., fearful response), cognitive transmission (e.g., interpretation bias), other parenting behaviours (e.g., over-control), or through an insecure attachment [21, 29].

In line with existing evidence on the risks of parental anxiety on child behaviour problems [10, 12], the current study found that children of mothers with higher anxiety levels at baseline were more likely to exhibit behaviour problems two years later. However, when mother’s anxiety level and baseline child behaviour problems were controlled for, there was evidence (albeit weak) that children of mothers who had engaged in more pretend play at baseline were less likely to exhibit behavioural problems at 2-year follow-up. These findings suggest that mothers’ engagement in pretend play might be a factor which prevents the development of behavioural problems in their children. One explanation is that mothers may support their children’s development of emotional regulation and social skills through engaging in pretend play [3, 28]. For example, when mothers pretend with their children, children may feel encouraged to explore different emotions and try out different emotional responses in a secure setting. This may help children understand their own and others’ emotions and learn emotional regulation strategies. By pretending together with their mothers, children can also learn how to communicate, negotiate, and cooperate with others. An additional explanation is that engaging in pretend play together helps to promote a positive mother–child relationship (e.g., secure attachment), which in turn reduces the risks of children developing behaviour problems in the long run. These explanations may hold true in mothers with high and low anxiety levels, as a lack of interaction effect between maternal anxiety and mother pretend play suggested that the relationship between mother pretend play and child behavioural problems did not vary according to mothers’ anxiety levels.

Although the current study did not indicate any effects of child age and gender on parent–child play, it is possible that the way caregivers play with their children changes over time. Most mothers in the current study demonstrated more frequent pretend play than their 2-year-old children, which can be expected developmentally and highlights the important role that parents play in modelling pretend play behaviours. With their cognitive, social, and emotional development, older children are likely to take more of a lead in their play with their caregivers. Other child characteristics, such as temperament, might also play a role in the parent–child interaction.

### Strengths

One strength of the current study is its direct examination of mothers’ and their children’s play behaviour when playing with a toy set together at home. The mother–child interaction was coded by researchers who were not involved in data collection and were blinded to participant characteristics and outcomes during coding, which reduced bias and enhanced the validity of the study. The coding, conducted at five-second segments for five minutes, allowed variations in patterns of play in mothers and children to be captured. Using a longitudinal design, the current study allowed the investigation of how mother pretend play at baseline predicted child behavioural problems at 2-year follow-up, when accounting for baseline child behavioural problems, baseline maternal anxiety and depression. This provides valuable insights into the potential influence of maternal play behaviour during children’s earliest years on their later outcomes, which is important for designing interventions aiming to reduce long-term child behavioural problems.

### Limitations

The current study uses a community sample of mothers, which might limit its generalisability to clinical samples. Additionally, findings from the current study might not be generalised to fathers, as research has suggested that mothers and fathers may interact with their children differently and influence child development in different ways [27]. Child behavioural problems were rated by mothers in the current study, which might have been biased given the possible influence of maternal anxiety or depression on mothers’ reports on children’s behaviour [5].

### Suggestions for future research

Future investigation is warranted to explore the potential effects of anxiety and/or depression on parent–child pretend play in both mothers and fathers, in high and low risk samples, and in parents with more or less chronic symptoms. A further direction is to examine anxiety and depression separately to better tease apart their associations with pretend play. Given that pretend play could be a feasible target for intervention, further research would be valuable to test more explicitly the role of mother pretend play as a factor that reduces the risks of child behavioural problems.

The current study focused on frequencies of pretend play in mothers and their children. It is possible that other characteristics of play (e.g., frequency of other forms of play and other features in pretend play such as pretend themes and quality of pretend play) are associated with maternal anxiety and depression. These warrant future investigation and could have implications for interventions.

## Conclusions

To conclude, the current study suggests that maternal anxiety and depression are associated with lower levels of pretend play in mothers and their children. It also indicates that higher levels of mother’s engagement in pretend play might serve as a factor which helps prevent the development of child behaviour problems. It highlights the importance of examining parent–child play to understand the possible mechanisms through which parental anxiety and depression influence child development. The current study has important implications for healthcare and community practices. It suggests that supporting mothers to engage in pretend play with their children could be a target for interventions aimed at families affected by maternal anxiety and depression. Supporting mother–child play might also be incorporated into interventions that aim to reduce long-term child behaviour problems.

## Data Availability

All of the individual de-identified participant data referenced in the study will be available 12 months following publication and for 5 years after date of publication. Data will be made available to researchers who provide a methodologically sound proposal and have the required institutional approvals in place, to achieve aims in the approved proposal. Proposals should be directed to Professor Paul Ramchandani pr441@cam.ac.uk to gain access and requestors will be asked to sign a data access agreement.

## Appendix

See Table

**Table 5:** Coding scheme for mother–child pretend play

Mother	Child	Description		Examples
Pretend play	Pretend play	Sound	Mimic the sound of an animal	Making the sound of a pig (or monkey, tiger) while holding a puppy pig (or monkey, tiger)
			Mimic the sound of a vehicle	Making the sound of a car while moving a toy car
			Mimic the sound of a person	Making the sound as if crying while at the same time smiling
		Speech	Suggest or narrate a pretend action	“Shall we pretend they are eating?”
				“This is our lunch”
			Suggest or narrate a pretend property	“Is it yummy?” (after play partner pretending to drink milk from an empty cup)
				“This (the toy sandwich) is tasty”
			Suggest or narrate an imaginary object	“What’s in your cup? juice?” (the cup is empty)
				“There is a monster”
			Use an obviously different voice to speak	Talking in a way as if it is another person speaking
		Behaviour	Use a puppy to act out pretend behaviour	Moving puppy in a way that a clear action (e.g., eating, drinking) is acted out
			Manipulate a toy to act out pretend behaviour	Pour a toy teapot as if pouring tea to the cup
				Use one’s toy cup to touch play partner’s toy cup as if cheering
			Use one’s own body to act out pretend behaviour	Moving one’s hands as if chopping/eating food
Not pretend	Not pretend	No clear pretend sound, speech or behaviour is observed		Putting puppy on fingers without talking or acting

5.

## Footnotes

1 Of the baseline sample (N = 60), 3 children did not complete the 2-year follow up study, therefore their scores on CBCL at 2-years follow-up were missing. These 3 children were excluded for all the analyses involving the 2-year follow-up CBCL scores.
